# Global cognitive function is associated with sex, educational level, occupation type, and speech recognition rate in older Chinese adults: a single-center, prospective, cross-sectional study

**DOI:** 10.1186/s12877-022-03603-5

**Published:** 2022-12-08

**Authors:** Hailing Gu, Xinyi Yao, Cong Diao, Min Liu, Weili Kong, Haotian Liu, Yu Zhao, Zhaoli Meng

**Affiliations:** 1 Department of Otolaryngology-Head and Neck Surgery, Hearing Center/Hearing and Speech Science Laboratory, West China Hospital of Sichuan University, 37 Guo Xue Lane, Chengdu, 610041 Sichuan People’s Republic of China; 2 Department of Otolaryngology, Head and Neck Surgery, West China Hospital, Sichuan University, Chengdu, China

**Keywords:** Age-related hearing loss, Mild cognitive impairment, Mini-mental state examination

## Abstract

**Background:**

The development of cognitive impairment may be delayed if its risk factors are identified and detected, if its developmental trend can be predicted, and if early intervention can be performed. This study primarily aimed to investigate the association between global cognitive function and hearing loss, educational level, and occupation type and to determine any differences in such associations according to sex among older Chinese adults.

**Methods:**

In this cross-sectional study, we prospectively recruited 219 individuals above 55 years old in an otolaryngology outpatient clinic who could write independently and had no severe vision impairment. Audiometric examinations included otoscopy, acoustic immittance, pure-tone audiometry, and speech audiometry for each ear. Cognitive function was evaluated by using the Chinese version of the Mini-Mental State Examination (MMSE). Multivariable linear regression analyses were performed to evaluate the relationship between variables and MMSE scores after adjusting for independent variables that were statistically significant in the univariable analyses.

**Results:**

We enrolled 219 individuals: 98 men (mean ± standard deviation age, 63.08 ± 6.64 years) and 121 women (62.64 ± 7.17 years). The overall MMSE scores of the normal hearing group and the mild, moderate, and severe-to-profound hearing loss groups were 24.00 (5.00), 24.00 (5.00), 23.00 (5.00), and 23.00 (13.00), respectively. MMSE scores were higher among participants with higher educational levels (*p* < 0.001) and were significantly correlated with occupation type (*p* < 0.001). MMSE scores were significantly higher in men than in women (*p* < 0.001). However, after the analysis of the five subdomains, significant differences were only observed for attention and calculation (*p* < 0.001) and language (*p* = 0.011). We further compared the distribution of educational levels between men and women by using the chi-square test; there was no significant difference in educational level between the sexes (*p* = 0.070).

**Conclusions:**

We reported statistically significant relationships between global cognitive function and sex, educational level, and occupation type. Sex-specific strategies may be required to improve healthcare policies.

## Background

Cognitive impairment (CI) affects the quality of life, social functioning, and well-being of older adults. Given that the number of older adults is increasing with the increase in average life expectancy, CI is becoming a severe social and public health problem. The prevalence of mild CI (MCI) reported in population-based epidemiological studies ranges from 3 to 19% in adults older than 65 years [1]. MCI can act as a transitional stage in the development of dementia with a range of conversion of 10–15% per year, but it does not interfere substantially with individuals’ daily activities [2]. Approximately 50 million people live with dementia worldwide, and this number is projected to increase to 152 million by 2050, particularly in low- and middle-income countries, where approximately two-thirds of people with dementia live [3].

In addition to the development of pharmacological preventions and treatments for CI, modifiable risk factors that may be associated with or contribute to CI need to be examined and addressed. Baltes and Lindenberger [4] proposed the cognitive load on perception hypothesis, which states that reduced cognitive capacity places a heavier load on perception, thus affecting sensory processing. Langa and Levine [5] identified the risk factors for cognitive decline, which include aerobic exercise, mental activity, and cardiovascular risk factors. Other studies indicated that age-related hearing loss (ARHL) is an independent risk factor [6, 7]. Depending on its severity, ARHL seems to play a significant role in cognitive decline, but the underlying mechanism of the relationship remains an open question. The type of hearing loss (HL) most commonly encountered in older adults is ARHL [8], and its clinical manifestation is speech perception difficulty in a noisy environment. Speech perception is a process in which people hear, interpret, and understand the sounds of language [9]. Instinctively, cognitive functions are involved in speech perception, but few studies have investigated their link.

A higher educational level and/or a complex occupational role is generally considered to protect against cognitive decline [10, 11] and to contribute to cognitive reserve, which helps the brain to actively cope with age-related changes and diseases via the flexibility and plasticity of cognitive networks [12]. Other putative protective factors may also be caused by a higher educational level and occupational complexity [13]. Dekhtyar et al. [14] indicated that a higher educational level decreased the risk of dementia, whereas occupational complexity was not associated with dementia. Furthermore, Dekhtyar et al. [14] and Jones et al. [15] revealed that the cut-off point at which educational level is protective against dementia may vary depending on the age composition of the cohort in question. Therefore, the influence of education and occupation type on cognitive performance in older adults is unclear.

Livingston et al. [16] reported that the prevalence of CI in China was 62.7% in women and 45.4% in men over 75 years of age. However, there are differences in hypotheses and results between studies regarding factors related to cognitive decline between the sexes among older individuals. Chou et al. [17] and Szoeke et al. [18] provided an explanation for the better cognitive performance of men than that of women, namely, the effects of endogenous estrogen decline at older age. Older women are more likely to develop dementia than men of the same age probably in part because, on average, older women have had less education than older men [5]. When adjusting for educational status, it seems that women typically have better verbal memory than men, and this is consistent with the cognitive reserve hypothesis [19]. However, the difference in cognitive performance between the sexes after adjusting for age remains unknown.

The development of CI may be delayed if its risk factors are identified and detected, if its developmental trend can be predicted, and if early intervention can be performed. The main aim of this study was to investigate the associations between global cognitive function (as defined by using Mini-Mental State Examination [MMSE] scores) and HL, educational level, and occupation type and to explore whether there are any differences in such associations according to sex among older Chinese adults.

## Methods

### Study population

In this cross-sectional study, we prospectively recruited individuals in the otolaryngology outpatient clinic of West China Hospital from June 2020 to February 2021.

Individuals were included in this study if they were aged 55 years or older [20], could write independently, and had no diagnosis of severe vision impairment. Participants were excluded if they were living alone [5]; had a history of more than 30 min of unconsciousness due to trauma [21]; had first-degree relatives who had been diagnosed with dementia; or had hypertension (systolic blood pressure ≥ 140 mmHg and/or diastolic blood pressure ≥ 90 mmHg) [22], diabetes mellitus (glycated hemoglobin > 6.5%) [23], hemorrhaging, cerebral infarction, cerebrovascular disease diagnosed with magnetic resonance imaging, body mass index > 24 or < 18.2 kg/m^2^, thyroid dysfunction, abnormal electrocardiogram characteristics, chronic obstructive pulmonary disease (COPD Assessment Test score ≥ 11) [24], syphilis, or unilateral/bilateral conductive/mixed HL. The aim of this criteria was to avoid confounding factors, obtain relatively rigorous results, and provide strong evidence of the scientific association between HL and cognitive function. Individual medical history (dementia, hypertension, hemorrhaging, cerebral infarction, cerebrovascular disease, etc.) was collected by using a pre-designed questionnaire, which was used to select eligible participants.

This study was approved by the Biomedical Research Ethics Committee of West China Hospital (no. 2020285). All participants voluntarily signed an informed consent form.

After strict observance of the inclusion and exclusion criterion, a total of 219 individuals were enrolled in this study.

### Audiometric examinations

A combination of otoscopy, acoustic immittance, pure-tone audiometry, and speech audiometry was conducted for each ear in a soundproof room with ambient noise < 30 dBA (A-weighted sound pressure level). Bilateral pure-tone hearing thresholds at frequencies of 0.25, 0.5, 1, 2, 4, and 8 kHz were measured at 5 dB increments in dB hearing level. Pure-tone threshold average (PTA) of the better ear for four frequencies (0.5, 1, 2, and 4 kHz) were adopted to define the participants’ degree of hearing according to guidelines published by the World Health Organization in 1997 [25]. The cut-off values for mild, moderate, severe, and profound HL were 25, 40, 60, and 80 dB hearing level, respectively. Speech recognition rate was determined using speech audiometry. The acoustic immittance test consisted of tympanometry and the acoustic reflex decay test and was used to measure the state of the middle ear and the function of the cochlear and facial nerves.

### Cognitive assessment

Cognitive function was evaluated using the Chinese version of the MMSE. The MMSE has a 30-point scale and is commonly used to screen individuals for CI, with high sensitivity and specificity [26]. It covers five cognitive domains: orientation, registration, attention and calculation, recall, and language. The diagnostic criteria for CI varied with the participant’s educational level. CI was defined as an MMSE score > 17 in the illiterate group, > 20 in the primary school group, and > 24 in the middle school level and above group [27].

### Covariate

The selected covariates included age, sex, occupation type, educational level, and HL duration. The specific occupation of the participants was acquired in face-to-face interviews and classified into mental labor, physical labor, retired, or none by researchers on the basis of the Professional Classification Dictionary of The People’s Republic of China [28]. Mental labor refers to professional, managerial, or administrative work that is usually performed in an office or other administrative environment. Physical labor refers to strenuous physical work or other types of work that demand physical exertion. Educational level was categorized as illiteracy, primary, junior, high school, senior, undergraduate, master, and PhD on the basis of the self-report.

### Statistical analysis

Continuous variables (age, HL duration, PTA, and speech recognition rate) are described as means and standard deviations. Categorical variables (binary variable: sex; multi-categorical variables: educational level and occupation type) are presented as numbers with percentages. Student’s t-test, one-way analysis of variance, the Mann–Whitney U test, and the Kruskal–Wallis test were used for the comparison of continuous variables among groups, and the chi-squared test or Fisher’s exact test was used for the comparison of categorical variables among groups. Univariable linear regression analyses were conducted to assess the relationship between each variable and the MMSE score. Multivariable linear regression analyses were performed to evaluate the relationship between variables and the MMSE score after adjusting for independent variables that were statistically significant in the univariable analyses.

All statistical analyses were performed with IBM SPSS Statistics for Windows version 25.0 (IBM Corp., Armonk, NY, USA) and GraphPad Prism 9.0 software (GraphPad Software, Inc., San Diego, CA, USA), and a two-sided *P*-value ≤0.05 was considered statistically significant.

## Results

### Demographic characteristics

Among the 219 individuals enrolled in this study, 98 were men (44.7%; mean age, 63.08 ± 6.64 years) and 121 were women (55.3%; mean age, 62.64 ± 7.17 years). With regard to HL, 64 (29.2%) participants had normal hearing, 70 (32.0%) had mild HL, 73 (33.3%) had moderate HL, and 12 (5.5%) had severe–profound HL (severe and profound HL groups were combined because of the small sample size). Participant age, HL duration, PTA, and speech recognition rate differed among the four groups. Individuals with worse hearing were typically older, had had HL for a longer time, had a higher PTA, and had a worse speech recognition rate than those with better hearing. However, there were no differences in sex, educational level, or occupation type among the groups. The specific sociodemographic and hearing characteristics of the participants are summarized in Table 1.

**Table 1:** Sociodemographic characteristics of all participants by degree of HL

Characteristics	Hearing-loss degree				***P*** Value
	Normal (***n*** = 64)	Mild (***n*** = 70)	Moderate (***n*** = 73)	Severe-profound (***n*** = 12)	
Age, mean (SD), years	59.92 (4.62)	63.23 (6.83)	64.42 (7.51)	66.42 (9.22)	**0.001**
Sex, n (%)					
Male	23 (23.47)	33 (33.67)	37 (37.75)	5 (5.10)	0.146
Female	41 (33.88)	37 (30.58)	36 (29.75)	7 (5.79)	
Education, n (%)					
Illiteracy	2 (16.67)	3 (25.00)	5 (41.67)	2 (16.67)	
Primary school	6 (23.08)	9 (34.62)	11 (42.31)	0 (0.00)	0.249
Junior/ high school	36 (29.51)	36 (29.51)	42 (34.43)	8 (6.56)	
Undergraduate/ master/ PhD	20 (33.90)	22 (37.29)	15 (25.42)	2 (3.39)	
Occupation type					
None	18 (29.03)	18 (29.03)	20 (32.26)	6 (9.68)	0.668
Retired	35 (32.71)	33 (30.84)	36 (33.64)	3 (2.80)	
Physical labor	6 (24.00)	9 (36.00)	8 (32.00)	2 (8.00)	
Mental labor	5(20.00)	10(40.00)	9(36.00)	1(4.00)	
Duration of HL, mean (SD), years	0.33(1.37)	1.87(4.47)	5.91(11.23)	13.21(15.15)	**0.000**
PTA, mean (SD), dB HL	15.94(4.71)	31.18(5.40)	49.38(5.78)	66.87(10.92)	**0.000**
Speech recognition rate, mean (SD), %	98.56(2.61)	94.57(10.64)	49.38(5.78)	66.88(10.92)	**0.000**

*HL* hearing loss, *SD* standard deviation, *PTA* pure-tone threshold average

### Primary outcome: association between cognition and hearing

**Fig. 1:**
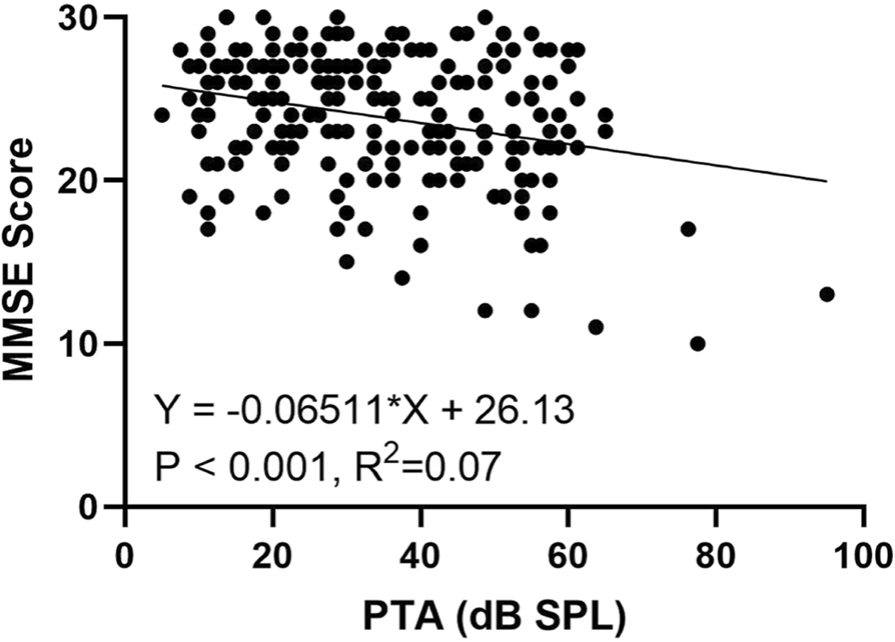
Relationship between PTA and MMSE score with linear regression

**Fig. 2:**
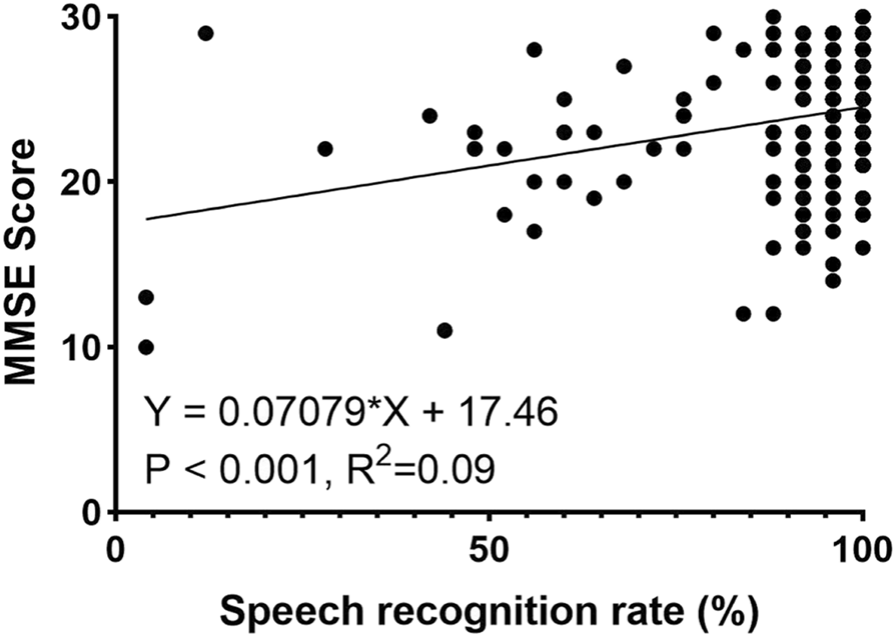
Correlation between speech recognition rate and MMSE score with linear regression

The overall MMSE scores of the normal hearing group and the mild, moderate, and severe–profound HL groups were 24.00 (5.00), 24.00 (5.00), 23.00 (5.00), and 23.00 (13.00), respectively. There was no significant association between MMSE score and the degree of HL (*p* = 0.097). Linear regression analysis revealed that the MMSE score was negatively correlated with PTA (R^2^ = 0.07, *p* < 0.001, Fig. 1) and positively related to speech recognition rate (R^2^ = 0.09, *p* < 0.001, Fig. 2).

### Secondary outcome: association between cognition and other covariates

#### Association between cognition and educational level

MMSE scores were higher among participants with higher educational levels (*p* < 0.001). The overall MMSE scores of the illiterate, primary school, junior/high school, and undergraduate/master’s/PhD groups were 17.50 (6.00), 20.50 (5.00), 24.00 (5.00), and 27.00 (4.00), respectively. The above trend among the four education levels was also present in the MMSE subdomains of orientation (*p* < 0.001), attention and calculation (*p* < 0.001), recall (*p* < 0.001), and language (*p* < 0.001) but not in registration (*p* = 0.142, Table 2).

**Table 2:** The difference in MMSE scores under the stratification of covariates

Characteristics	MMSE Score, median (interquartile range)											
	Total	***P***	Orientation	***P***	Registration	***P***	Attention and calculation	***P***	Recall	***P***	Language	***P***
Age												
55 ~ 59 years	24.00(5.00)	0.495	8.00(2.00)	0.255	3.00(0.00)	0.673	5.00(4.00)	0.991	2.00(3.00)	0.481	9.00(1.00)	0.345
60 ~ 64 years	24.00(5.00)		8.00(2.00)		3.00(0.00)		5.00(3.00)		2.00(3.00)		9.00(1.00)	
65 ~ 69 years	25.00(4.00)		8.00(2.00)		3.00(0.00)		5.00(3.00)		2.00(3.00)		9.00(1.00)	
≥ 70 years	24.00(6.00)		7.00(2.00)		3.00(0.00)		5.00(4.00)		2.00(3.00)		9.00(2.00)	
Sex												
Male	25.50(5.00)	**< 0.001**	8.00(2.00)	0.091	3.00(0.00)	0.985	5.00(2.00)	**< 0.001**	2.00(2.00)	0.128	9.00(1.00)	**0.011**
Female	23.00(5.00)		8.00(1.00)		3.00(0.00)		3.00(4.00)		2.00(2.00)		9.00(1.00)	
Education												
Illiteracy	17.50(6.00)	**< 0.001**	6.50(3.00)	**< 0.001**	3.00(0.00)	0.142	1.50(2.00)	**< 0.001**	0.00(0.00)	**< 0.001**	6.00(2.00)	**< 0.001**
Primary school	20.50(5.00)		6.50(2.00)		3.00(0.00)		2.00(3.00)		0.00(2.00)		8.00(3.00)	
Junior/ high school	24.00(5.00)		8.00(1.00)		3.00(0.00)		5.00(3.00)		2.00(1.00)		9.00(1.00)	
Undergraduate/master/ PhD	27.00(4.00)		8.00(1.00)		3.00(0.00)		5.00(1.00)		2.00(2.00)		9.00(0.00)	
Occupation type												
None	23.00(6.00)	**< 0.001**	7.00(2.00)	**< 0.001**	3.00(0.00)	0.374	4.50(4.00)	**0.003**	1.00(2.00)	**0.001**	8.00(2.00)	**< 0.001**
Retired	25.00(5.00)		8.00(2.00)		3.00(0.00)		5.00(3.00)		2.00(2.00)		9.00(1.00)	
Mental labor	27.00(4.00)		8.00(1.00)		3.00(0.00)		5.00(0.00)		2.00(2.00)		9.00(1.00)	
Physical labor	22.00(7.00)		7.00(3.00)		3.00(0.00)		3.00(4.00)		1.00(2.00)		8.00(2.00)	
Hearing Loss degree												
Normal	25.00(5.00)	0.097	8.00(1.00)	0.130	_	**< 0.001**	4.00(3.00)	0.700	2.00(2.00)	0.175	9.00(1.00)	0.080
Mild	25.00(5.00)		8.00(2.00)		3.00(0.00)		5.00(3.00)		2.00(3.00)		9.00(1.00)	
Moderate	23.00(5.00)		8.00(1.00)		3.00(0.00)		5.00(4.00)		2.00(3.00)		9.00(1.00)	
Severe-profound	23.00(13.00)		7.50(2.00)		3.00(3.00)		5.00(4.00)		1.00((2.00)		8.50(2.00)	

#### Association between cognition and occupation

MMSE scores were significantly correlated with occupation type (*p* < 0.001). The overall MMSE scores of the unemployed, retired, mental labor, and physical labor groups were 23.00 (6.00), 25.00 (5.00), 27.00 (4.00), and 22.00 (7.00), respectively. The distribution of the overall MMSE score and those for different occupation type is illustrated in Table 2.

#### Association between cognition and sex

MMSE scores were significantly higher in men than in women (*p* < 0.001). However, upon analysis of the five subdomains, significant differences were only observed for attention and calculation (*p* < 0.001) and language (*p* = 0.011). Scores did not differ between sexes in terms of orientation (*p* = 0.091), registration (*p* = 0.985), or recall (*p* = 0.128) (Table 2). We further explored whether the educational level is related to the cognitive differences between the sexes by comparing the distribution of educational levels between men and women by using the chi-squared test. This analysis revealed that there was no significant difference in the educational level between the sexes (*p* = 0.070, Table 3).

**Table 3:** The distribution difference in educational level between males and females

Sex, n (%)	Education level				***P***-value
	Illiteracy	Primary school	Junior/ high school	Undergraduate/ master/ PhD	
Male	2(2.04)	9(9.18)	55(56.12)	32(32.65)	0.070
Female	10(8.26)	17(14.05)	67(55.37)	27(22.21)	

### Tertiary outcome: association of MMSE score with all measured characteristics

Table 4 summarizes the results of the linear regression analyses. In the univariable analysis, the MMSE score was significantly associated with sex, educational level, occupation type, HL duration, PTA, and speech recognition rate (all *p* < 0.05). In the multivariable analysis, the HL duration (*p* = 0.794) and PTA (*p* = 0.212) were excluded from the model. The MMSE scores of retired participants and those with a physical job did not differ from those of unemployed participants (*p* = 0.872 and *p* = 0.239, respectively).

**Table 4:** Association of MMSE score with measured characteristics in the simple and multiple linear regression analysis

Characteristics	Univariate analysis			Multivariate analysis		
	β	95% CI	***P*** Value	β	95% CI	***P*** Value
Age	−0.06	−0.14, −0.01	0.096	−0.027	–	0.612
Sex						
Male	Reference			–	–	–
Female	−1.82	−2.85, −0.79	**0.001**	−1.30	−2.11, −0.50	**0.002**
Education						
Illiteracy	Reference			–	–	–
Primary school	3.68	1.54, 5.82	**0.001**	2.79	0.73, 4.85	**0.008**
Junior/ high school	7.49	5.63, 9.35	**< 0.001**	6.46	4.67, 8.23	**< 0.001**
Undergraduate/master/ PhD	9.638	7.69, 11.58	**< 0.001**	8.13	6.24, 10.02	**< 0.001**
Occupation type						
None	Reference					
Retired	2.94	1.82, 4.07	**< 0.001**	0.01	–	0.872
Physical labor	4.28	2.60, 5.96	**< 0.001**	0.07	–	0.239
Mental labor	−0.48	−2.16, 1.20	0.573	−1.92	−3.17, − 0.66	**0.003**
Duration of hearing loss	−0.08	−0.14, 0.01	**0.02**	−0.01	–	0.794
PTA	−0.07	−0.10, 0.03	**< 0.001**	− 0.08	–	0.212
Speech recognition rate	0.07	0.04, 0.10	**< 0.001**	0.05	0.03. 0.08	**< 0.001**

*HL* hearing loss, β regression coefficient, *CI* confidence interval, *PTA* pure-tone threshold average

## Discussion

Our study aimed to investigate the relationship between HL, educational level, occupation type, and cognition function among older Chinese adults and whether such associations differ according to sex. By using univariate analysis, we identified six factors associated with CI: sex, educational level, occupation type, HL duration, PTA, and speech recognition rate. After adjusting for covariates, we found that the MMSE score was lower among female participants, those with a lower educational level, those with mental labor occupations, and those with a higher speech recognition rate than among other groups.

We have demonstrated that severe HL is associated with an increased risk of developing CI, which manifests as a lower MMSE score. Fetoni et al. [29] compared patients with and without cognitive dysfunction by using MMSE and discovered a higher hearing threshold in those with cognitive dysfunction (*p* = 0.049). In Guglielmi et al. [30], HL affected episodic memory and attentional functions rather than executive functions. In fact, there is a large consensus that ARHL is an independent and modifiable risk factor for cognitive decline [31, 32]. It is widely accepted that auditory deprivation triggers a vicious circle in older people involving social isolation and CI [33, 34]. Excessive cognitive load dedicated to auditory perceptual processing in everyday life causes structural changes in the brain and neurodegeneration, which are detrimental to other cognitive processes [35, 36]. We combined subjective measures and objective audiometry in the current study and discovered that an increase in HL severity and a poorer speech recognition rate were associated with a lower total MMSE score. Difficulties in understanding language in older adults result from age-related defects in peripheral and central auditory pathways because they are more likely to have decreased cochlear blood supply and loss of outer hair cells at the cochlear basal [37].

Our research also revealed a statistically significant correlation between educational level and cognitive function in four of the five MMSE subdomains (all except registration) even after adjusting for confounding factors. In the registration subdomain, participants are scored on the repetition of three words (namely, “tree,” “clock,” and “car”) on the first attempt after the words were read out to them. When sufficient auditory stimuli were presented, all three words were typically repeated correctly irrespective of the participant’s educational level. Consistent with a Brazilian community sample, education also did not have an important effect on memory registration [38]. However, overall MMSE scores are highly dependent on educational level: low education levels may affect the understanding of MMSE tests [39–41]. Furthermore, with increasing educational duration, the levels of MMSE scores increase. In a study of community-dwelling older adults aged 60 years or older, a higher educational level was associated with better cognitive function [42]. Individuals with hearing impairment need to devote greater resources to understanding poor-quality auditory signals, thus leaving insufficient resources available for other cognitive activities; a higher educational level may provide sufficient cognitive reserve to counteract the effects of mild hearing impairment [43].

Our results support the findings of Stern et al. [10], who indicated that occupation type was related to cognitive function and may influence cognitive health via physiological and psychological pathways. Job stress may be a potential modifiable risk factor for adverse cognitive outcomes. In an epidemiological catchment area study in Baltimore, low-strain jobs were associated with statistically significantly lower decreases in cognitive scores than other job groups over an approximately 11-year period [44]. Normally, stress responses help individuals deal with urgent situations by activating the hypothalamus–pituitary–adrenal axis and increasing cortisol levels [45]. Given that a sense of low control is associated with high psychological stress, people with low job control bear higher risks of cognitive decline when facing higher job demands [46]. Furthermore, a higher educational level, higher cognitive level of a person’s occupation (white collar jobs, e.g., clerical work, medical practice, and other occupations requiring a university degree), and greater engagement in cognitive leisure activities were reportedly related to higher MMSE scores [47]. These results support the view that cognitive stimulation throughout the course of a person’s life may contribute to cognitive reserve, thereby protecting that person against cognitive decline [48–50].

Across our cohorts, men generally performed better than women for the MMSE subdomain of attention and calculation (but not the other subdomains) after adjusting for educational level. This result complements that of a report by the Lancet Commission [5]. However, only a few studies [51, 52] have demonstrated the presence of sex differences in MCI. Better attention and calculation performance in men than in women could arise from the effect of estrogen [53] or sex-specific cognitive reserve [5]. An eight-year longitudinal study revealed that cognitive deterioration in women with MCI was twice as fast as that in their male counterparts [54]. Even with the same degrees of hippocampal atrophy and the same rates of glucose metabolism in the temporal lobe, verbal memory performance reportedly differs between male and female patients with MCI [55]. The brain atrophy rate of female patients with MCI is reportedly higher than that of male patients, with an additional decrease of 1.0–1.5% per year [56]. A possible explanation is that men may have higher resilience to MCI-related pathological damage to the brain [51] and better executive function than women; this finding is supported by cognitive reserve theory. However, this is different from the results of a previous study [57]. The reason for this difference may be due to population heterogeneity because the relationship between ARHL and CI was stronger in the population of the current study. Furthermore, another possible explanation is the different cognitive function assessment tools used and the different definitions of HL. This study identifies the need for sex-specific strategies in the risk factor modification and healthcare prevention policies of MCI in adults above 55 years. Beyond traditional risk factors, gender-related characteristics, such as psychological stressors, also play a sizable role in MCI risk; hence, a multifaceted approach targeted to these risk factors is important to improve sex-based differences in patient outcomes.

The main strength of our study is the strictness of the inclusion and exclusion criteria, which may have reduced potential confounders. Another major strength was our ability to adjust for educational level to allow the comparison of sex differences among older adults because educational level is highly correlated with MCI. However, there were also several limitations to our study. First, our results were based on cross-sectional data rather than longitudinal trajectories of HL and cognitive function. Second, this study was only a single-center study; therefore, the results had limited reproducibility for older individuals. Third, we assessed cognitive function mainly with the MMSE. Future research should be focused on a more comprehensive approach for the assessment of cognition, and longitudinal studies are needed to better explore the possible causal relationships between HL and cognitive function.

## Conclusions

In this study, we reported statistically significant relationships between global cognitive function as defined via the MMSE score, sex, educational level, occupation type, and speech recognition rate. Sex-specific strategies may be required to improve healthcare policies. These results indicate that speech recognition rate may be associated with CI among older Chinese individuals, who should be screened routinely for the early identification of the risk of cognitive decline. Our results need to be confirmed with prospective, longitudinal cohort studies.

## Data Availability

The datasets generated and/or analyzed during the study are not publicly available to protect patient privacy but are available from the corresponding author upon reasonable request.

## Abbreviations

ARHL: Age-related hearing loss
CI: Cognitive impairment
HL: Hearing loss
MCI: Mild cognitive impairment
MMSE: Mini-Mental State Examination
PTA: Pure-tone threshold average
